# Cancer incidence and mortality in Medellin-Colombia, 2010-2014

**DOI:** 10.25100/cm.v49i1.3740

**Published:** 2018-03-30

**Authors:** Mary Ruth Brome Bohórquez, Diego Mauricio Montoya Restrepo, Liseth Amell

**Affiliations:** Registro Poblacional de Cáncer de Antioquia. Secretaría Seccional de Salud y Protección Social de Antioquia, Medellin, Colombia

**Keywords:** Incidence, Mortality, Cancer, Medellin, Colombia, Incidencia, Mortalidad, Cáncer, Colombia

## Abstract

**Background:**

This study provides information on cancer incidence and mortality in a Colombian population during 2010-2014, based on the data and methodology of the Population-based Cancer Registry of Antioquia to facilitate the implementation of cancer control strategies.

**Methods:**

This is a descriptive study of cancer incidence and mortality in a population, residing in the urban area of the municipality of Medellin. The cancers included in the study are those prioritized in the cancer control plan for Colombia (PDCC-cancers). The collection, processing and systematization of the data were performed in accordance with internationally standardized parameters for population cancer registries. Incidence and mortality rates were calculated by gender, age and tumor location.

**Results:**

During 2010-2014 there were 22,379 new cancer cases recorded in the urban area of the municipality of Medellin, of which 43.5% corresponded to the PDCC-cancers. During the same period, 14,922 cancer deaths were reported, 23.5% related to the PDCC-cancers, 53.5% in women. Prostate cancer and breast cancer were the principal cause of morbidity in men and women, respectively, and lung cancer was the principal cause of death for both sexes.

**Conclusion:**

Cancer is a health problem for the population of Medellin. It is necessary to emphasize research and monitor risk factors, the health response and the capacity of the health provider network when facing the growing demand caused by this epidemic.

## Resumen

**Antecedentes:**

Es un estudio sobre la incidencia y mortalidad por cáncer en una población colombiana (2010-2014), a partir de los datos y la metodología del Registro Poblacional de Cáncer (RPC) de Antioquia para facilitar la implementación de estrategias para el control del cáncer.

**Métodos:**

Estudio descriptivo, cuya población objeto reside en el área urbana del municipio de Medellín. Se incluyeron para el análisis los cánceres priorizados por el plan de control de cáncer de Colombia (cánceres-PDCC). La recolección, el procesamiento y la sistematización de los datos se realizaron de acuerdo con los parámetros estandarizados internacionalmente para los RPC. Las tasas de incidencia y mortalidad se calcularon por sexo, edad y ubicación del tumor.

**Resultados:**

En el periodo 2010-2014 se registraron 22,379 casos nuevos de cáncer en el área urbana del municipio de Medellín, 43.5% corresponde a los cánceres-PDCC. Para el mismo periodo se reportaron 14,922 muertes por cáncer, 23.5% pertenecen a cánceres-PDCC, 53.5 %, ocurrieron en mujeres. El cáncer de próstata y el cáncer de mama fueron la primera causa de morbilidad por cáncer en hombres y mujeres respectivamente, y el cáncer de pulmón fue la principal causa de muerte por cáncer para los dos sexos.

**Conclusión:**

El cáncer es un problema de salud para la población del municipio de Medellín. Se requiere hacer énfasis en las investigaciones y monitoreo de los factores de riesgo, así como hacer seguimiento a la respuesta sanitaria y la capacidad de la red de prestadores frente a la demanda creciente de esta epidemia.

## Introduction

Cancer is one of the principal causes of morbidity and mortality around the world; in 2015 there were 8.8 million deaths. It is estimated that the number of new cases will increase by approximately 70% in the next 20 years ^1^ ^,^ ^2^. In Colombia from 2007 to 2011 there were an estimated 29,734 new cancer cases (men) and 33,084 (women) per year. The age standardized incidence rate (ASIR) for every cancer, except skin, per 100,000 inhabitants was 151.5 in men and 145.6 in women ^3^. 

In response, the Ministry of Health and Social Protection and the National Cancer Institute (or INC) implemented the *Model for Cancer Control in Colombia* in 2006. This model defines cancer control as a series of activities that seek to decrease the burden of the illness in Colombia. In order to execute the plan, a description of the epidemiological situation and an evaluation of the determinant factors is fundamental, which allows the health system to orient oncological service and ensure an adequate social response ^4^. The Decade Cancer Control Plan (or PDCC) ^5^ focuses its actions on the control of breast, cervix, prostate and colorectal cancer as well as acute pediatric leukemia. These tumors, in addition to stomach cancer, correspond to 31.7% of cancer mortality in Colombia during 2007-2011^3^.

Since 2000 the mission of the Population-based Cancer Registry of Antioquia (or RPC-A) is to offer reliable and high-quality cancer data in the state of Antioquia and facilitate the implementation of prevention and diagnostic programs and integral cancer care to diminish the burden of this disease in the region. Currently, the RPC-A is part of the International Association of Cancer Registries (or IACR) and the Network of Population-based Cancer Registries of Colombia, alongside the RPC of Cali, Bucaramanga, Pasto, Manizales and Barranquilla.

The present study aims to describe the cancer incidence and mortality in Medellin during 2010-2014, as both a contribution from the RPC-A to understand better the epidemiological behavior of cancer in the state of Antioquia and to come closer toward achieving the objectives outlined in the PDCC. 

## Materials and Methods

Medellin is the second most populated city in Colombia, capital of the state of Antioquia. The city is situated in the middle of the Central Mountain range of the Andes, 1,538 meters above sea level. The weather is warm with little variation in temperature throughout the year. The Medellin River runs through the entire length of the city. It is the nucleus of the metropolitan area in the Aburra Valley. The other neighboring municipalities from north to south are Barbosa, Girardota, Copacabana, Bello, Envigado, Itagüí, Sabaneta, La Estrella and Caldas. Industry represents 43.6% of the internal product. The industrial sectors in order of economic participation are the textile industry (20%), chemical products and substances (14.5%), beverages (11.0%) and food (10.0%). The remaining 10.0% include sectors such as metal mechanics, electrical and electronic industries, among others ^6^. 

### Population

**Figure 1:**
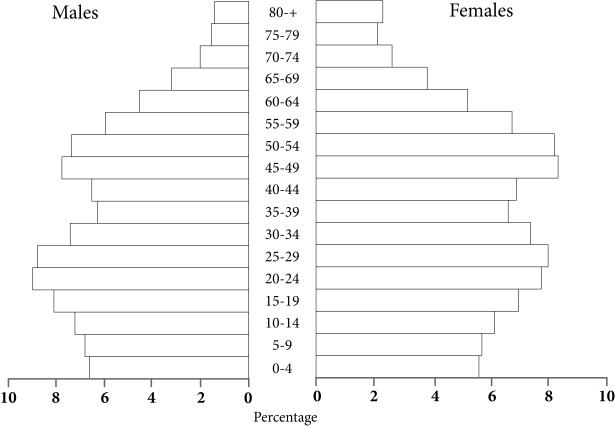
Medellin, Colombia. Population pyramid, 2013. Source: Based on projections from the National Administrative Department of Statistics. DANE, Colombia, 2013.

The inhabitants descend from a mixture of European, Indigenous and African origin, with a clear preponderance of the first. According to estimates from the National Administrative Department of Statistics in Colombia (or DANE), in 2013 there were 2,386,233 inhabitants in the urban area and 31,092 in the rural area, in total 52.9% of the population were women (Fig. 1). The Aging Index jumped from 8 senior citizens per 100 minors below the age of 15 in 1964 to 51 seniors in 2013; life expectancy increased from 60 years in the mid-20th century to 77.5 years in 2013, 75.5 years for men and 78.7 years for women ^7^.

### Registry area

The state capitol of Medellin is comprised of 105 km^2^ of urban area and 270 km^2^ of rural area.

### Case definition

For this study the RPC-A acts as a selective population registry. It included all of the malignant, invasive tumors diagnosed for the first time in the five year span between 2010-2014 (incidence) by the locations prioritized in the strategic lines of the PDCC in Colombia 2012-2020: breast (in women), cervical, prostate, colorectal and acute pediatric leukemia, in addition to stomach cancer. The basis of diagnosis could be histological, clinical, bone marrow cytology or death certificate. The statistical analyses only included primary malignant tumors or multiple invasive. In the analysis pre-invasive cervical neoplasms will be taken into account. Benign tumors and those of uncertain behavior were excluded. The cases of patients that arrived to the city for diagnosis and treatment were not considered residents of Medellin. The incidence date corresponds to the first chronological event of diagnosis, confirming the sickness. 

The information was collected by different means (active and passive), continuous and systematic recollection in the health institutes that produce cancer data: hospitals, clinics, oncology units, pathology and hematology laboratories, medical centers, specialized practices, the DANE and the State Secretary of Health and organisms in charge of processing death certificates, which provide the base to tabulate official mortality. The data collected were correlated to the patient’s socio-demographic variables, tumor clinic and follow up. The information was then processed according to the confidentiality criteria stipulated by the IACR for population-based cancer registries ^8^ ^,^ ^9^.

The cases were entered into the system designed by the RPC-A to eliminate duplicates, process and complement the data. The identification of multiple primary tumors follows the norms established by the IACR ^10^. Tumor topography (localization) and morphology (histology) were codified with the International Classification of Diseases for Oncology Third Edition (or CIE-O-3) ^11^. In order to compare the data, some localizations were grouped together. 

To ensure quality the RPC-A employs a set of indicators, exhaustiveness indicators (percentage of incident cases identified by death certificate and cause of death/incidence) and validity indicators (distribution of cases according to the most valid base for a cancer diagnosis and percentage of microscopically verified cases). 

The rates of incidence and mortality are calculated conventionally, utilizing the mid-year population estimates and projections denominator calculated by the official 2005 Census ^12^. Cases without age (0.5% 111/22,379) and *in situ* tumors were excluded from the study. In the case of cervical cancer, the incidence rates for invasive and pre-invasive neoplasms were calculated separately. The age adjusted rates were estimated by direct methods with the world population standard (or SEGI), the specific rates were calculated by variable: gender, localization and five-year age ranges (18 categories). The incidence and mortality results are presented for the 2010-2014 time period in their corresponding tables and graphs. 

## Results

From 2010 to 2014, 56,650 new cancer cases were registered in the RPC-A, (60.1% in women); and 30,465 cancer deaths were certified in the state of Antioquia (48.4% in men). Forty-five percent of the cases and 48.9% of cancer deaths occurred to permanent residents in the municipality of Medellin. 

Of the 22,379 new cancer cases diagnosed in Medellin, 61.6% were women. The average age for cancer diagnosis was 63 for men and 55 for women. The specific rates, crude rates (CR) and age standardized rates (ASR) with the world population are expressed per 100,000 people-year (p-y). For men the cancer incident rate per 100,000 people per year (p-y) for all localizations was 171.3 (CR) and 144.4 (ASR). In women the CR was 202.9 and the ASR 145.6.

Among men the five principle cancer localizations were prostate (26.9%), colorectal (7.8%), stomach (7.3%), lung (6.1%) and bladder (6.1%). Among women the most frequent localizations were: breast (25.8%), thyroid (13.6%), colorectal (7%), cervix (5.5%) and lung (4.4%). In all, these localizations represented 54.7% of all the new cancer cases diagnosed during the five-year period. 

Of the 14,922 cancer-related death certificates issued in Medellin, 53.4% were for women. The average age at the moment of death was 68 for men and 67.2 for women. Among men the cancer mortality rates per 100,000 p-y for all localizations was 123.3 (CR) and 101.1 (ASR). Among women the CR was 126.0 and the ASR was 82.6. The age specific cancer incidence and mortality rates were higher in women below 55 years of age, while for men it was after 55 (Supplementary Tables 1S and 2S).

Lung cancer (19%) was the first cause of death among men, followed by stomach cancer (11.5%), prostate (11.5%), colorectal (8.3%) and liver (6.2%). Lung cancer was the principle cause of death among women (15.1%), followed by breast cancer (13.5%), stomach (8.3%), colorectal (8.0%) and leukemia/lymphomas (7.6%). In all, these five principle causes constitute 52.5% of all cancer deaths that occurred in Medellin during the five-year interval from 2010 to 2014. 

### Cancers prioritized in the decade Cancer Control Plan in Colombia, 2012 - 2021. 

Of the total new cancer diagnoses in residents of Medellin, 9,538 (42.6%) corresponded to stomach, prostate, breast, cervical, colorectal cancer and acute pediatric leukemia, that altogether will be denominated for the analysis as PDCC-cancers; 4,075 (42.7%) cases were diagnosed in men and 5,463 (57.3%) in women.

For the PDCC-cancer group the CR and the ASIR per 100,000 p-y was 72.3 and 60.9 in men and 86.3 and 61.3 in women. 

During the 2010-2014 period there were 2,254 women with pre-invasive cervical lesions, in 44 cases the age was unknown, CR was 35.6 and the ASR 30.5.

### Quality criteria for incidence data

Table 1 shows the quality indicators for the incidence information during 2010-2014 period for malignant tumors prioritized in the PDCC. The percentage of diagnosed cases with microscopic verification (histology of primary tumor, cytology and bone marrow aspiration) for men and women was 99.8%; in this tumor group the percentage of registered cases that only had a death certificate as its only evidence was 0.2%. The global cause of mortality: incidence was 0.67 for the total population, 0.72 for men and 0.62 for women. 

**Table 1:** Municipality of Medellin, Colombia. Incidence and mortality data for malignant tumors prioritized by the Decade Cancer Control Plan in Colombia, quality indicators (exhaustiveness and validity) distributed by sex and localization during 2010-2014. Incident quality indicators.

Localization	Incidence					M:I	MV%	DCO%	Mortality			
	n	%	Age Desc	Rate					n	%	Rates	
				CR	ASR						CR	ASR
Breast (C50)	3,286	25.7	14	51.9	36.5	0.33	97.4	0.1	1,075	13.5	17.0	12.9
Cervix (C53)	708	5.5	1	11.2	8.5	0.55	98.0	0.1	391	4.9	6.2	4.9
Prostate (C61)	2,571	26.8	15	45.7	38.6	0.31	99.3	0.2	798	11.5	14.2	14.6
Colorectal (C18-C20)	1,640	7.3	5	13.7	10.0	0.74	98.5	0.2	1,216	8.1	10.2	7.2
Men	747	7.8	3	13.3	11.0	0.77	98.4	0.0	575	8.3	10.2	8.4
Women	893	7.0	2	14.1	9.4	0.72	98.6	0.3	641	8	10.1	6.5
Colon (C18)	1,080	4.8	3	9.0	6.6	0.94	98.6	0.2	1,015	6,8	8.5	6.0
Men	474	4.9	3	8.5	7.0	1.00	98.5	0.0	476	6,9	8.5	6.9
Women	606	4.7	0	9.6	6.4	0.89	98.7	0.3	539	6,8	8.5	5.4
Rectum (C19-C20)	560	2.5	2	4.7	3.4	0.36	98.8	0.2	201	1,3	1.7	1.2
Men	273	2.8	0	4.8	4.0	0.36	98.8	0.0	99	1,4	1.8	1.5
Women	287	2.2	2	4.6	3.0	0.36	99.3	0.3	102	1,3	1.6	1.1
Stomach (C16)	1,209	5.4	12	10.2	10.1	1.21	98.3	0.4	1,467	9,8	12.3	10.3
Men	695	7.2	6	12.4	12.3	1.15	98.4	0.3	801	11,5	14.2	13.6
Women	514	4.0	6	8.2	8.1	1.30	98.1	0.6	666	8,3	10.5	8.0
All localizations‡	22,379		111	187.1	143.4	0.67	96.3	0.2	14,922		124.7	89.7
Men	9,602	42.9	43	171.3	144.4	0.72	97.4	0.2	6,941		123.3	101.1
Women	12,777	57.1	68	202.9	145.6	0.62	95.5	0.2	7,980		126.0	82.6
Infantile Leukemia	77		0	0.6	1.1	0.60	98.7	1.3	46		0.4	0.6
Men	38		0	0.7	1.0	0.55	98.8	1.2	21		0.4	0.5
Women	39		0	0.6	1.1	0.64	98.6	1.4	25		0.4	0.7

**Source:** Population-based Cancer Registry of Antioquia. Vital Statistics SSSA - DANE.

**n**: number of cases;

**M:I**: Reason Mortality-Incidence;

**MV**: Verified Microscopically (histology/hematology);

**DCO:** Death Certificate Only. The specific, crude (CR) and age standardized rates (ASR) with the world population are expressed per 100,000 p-y.

‡ All localizations, except C44 (Skin non melanoma)

**Breast Cancer:** The average age of diagnosis was 58 years old, 42.5% in people below 50 years of age and only 12.5% over 80. 

**Figure 2:**
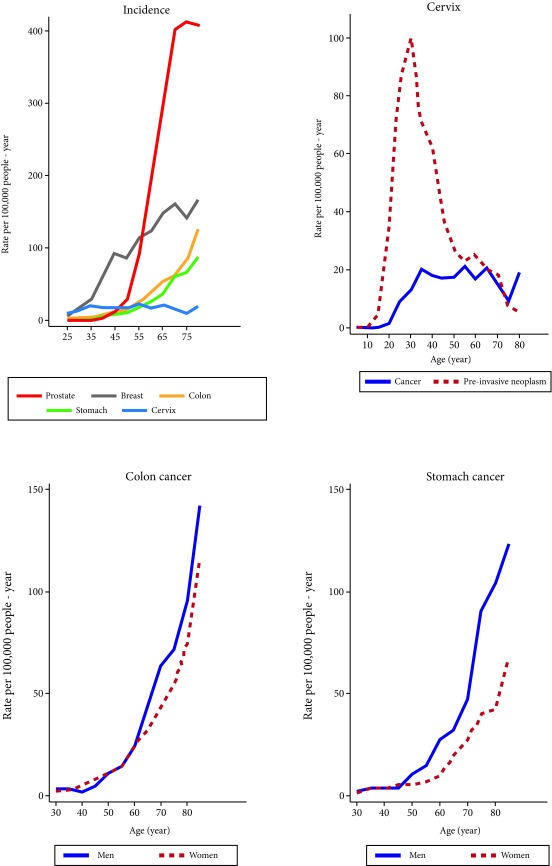
Medellin, Colombia, 2010-2014. Age and Sex specific incidence rates (per 100,000 p-y) for the 9,538 cancers prioritized in the PDCC in Colombia. **Source:** Population-based Cancer Registry of Antioquia. **A.** The age specific incidence rates were higher for prostate cancer and lower for cervical cancer. The incidence of precursor lesions of cervical cancer peaked at approximately 40 years of age. **B.** The morbidity of gastric cancer was greater for men older than 45 years old, the difference was less apparent for colorectal cancer with a slightly higher rate in women below 50 years of age.

**Cervical Cancer:** The specific incidence rates of invasive cervical cancer reached a maximum value of 20 per 100,000 at approximately 30 years of age and later leveled off and remained stable for all age groups, half of the cases were diagnosed before 50 and only 4.3% over 80 (Fig 2).

**Pre-invasive Cervical Neoplasms:** The peak incidence rate for pre-invasive cervical lesions occurred between 35 and 39 years old, 60.8% were diagnosed before 40. 

**Prostate cancer:** The average age of diagnosis was 68, 65 for colorectal cancer, 65 for stomach cancer and 7 for acute pediatric leukemia (Fig 2).

### Mortality by PDCC malignant tumors in Medellin

During 2010-2014, the PDCC-cancers represented 33.5% (4,993) of the total cancer deaths in Medellin (14,922); 2,798 (56.0%) of the deaths occurred in women and 2,195 (44.0%) in men.

The crude and standardized mortality rate (CMR and ASMR) by age for the PDCC-cancers was 38.9 and 31.6 in men and 44.2 and 29.7 in women. 

The average age at the moment of death was 64 for breast cancer, 58 for cervical cancer, 78 for prostate cancer, 68 for colorectal and stomach cancer. In the case of acute pediatric leukemia, the average age of death was 9. 

**Figure 3:**
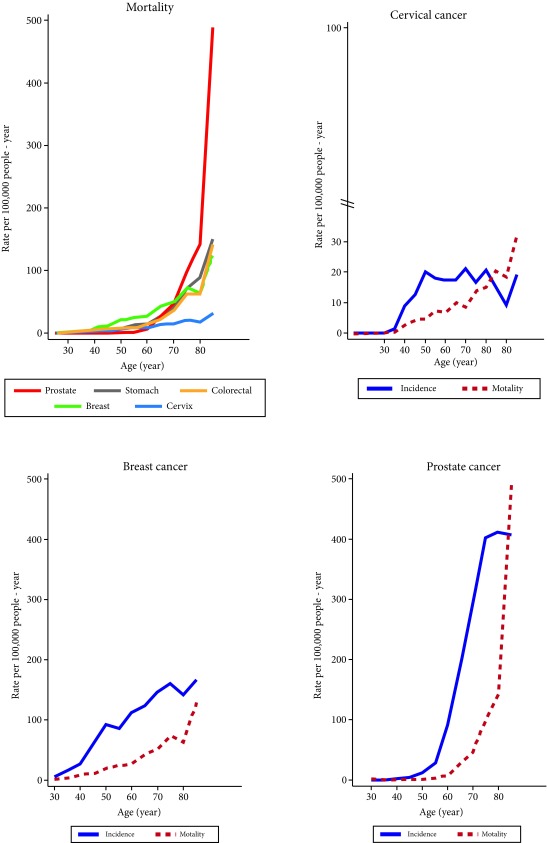
Medellin, Colombia, 2010-2014. Age and sex specific mortality rates (per 100,000 p-y). **Source:** Population-based Cancer Registry of Antioquia and Vital Statistics SSSA - DANE.

Figure 3 describes the curves for age specific mortality rates. The mortality rate for breast cancer is higher for women below 65 years old. For older adult older than 65 years of age deaths are caused by prostate and stomach cancer. This is the reason why (M:I) is greater than 1 in women with cervical cancer and in men with prostate cancer over 70. 

The specific mortality rates below 70 are higher in women with breast cancer. After 70 this is the reason why (M:I) is greater than 1 in men with prostate cancer and women with cervical cancer

## Discussion

The Population-based Cancer Registry of Antioquia (or RPC-Antioquia) compiled and classified all of the new cancer cases and cancer-related deaths that occurred in Medellin, the second most populated city in Colombia. This article presents the incidence and mortality rates for those cancers prioritized in the PDCC during 2010-2014. The PDCC-cancers were responsible for 42.4% of the morbidity and 33.4% of the cancer-related mortality in Medellin. This article is the product of a collaboration between the Secretary of Health of Antioquia and the network of oncological service providers in the city. 

The information provided by the RPC-A indicates that cancer is a public health problem in Medellin. It was the second cause of death after circulatory system diseases and responsible for 25% of deaths that occurred in the city ^13^. During the 5-year period (2010-2014) cancer was diagnosed in 5,225 new cases and 2,963 deaths a year for residents in the city of Medellin, according to the information provided by the Sectional Secretary of Health and Social Protection of Antioquia. 

The morbimortality risk for cancer in the region is determined by multiple factors. Cancer is part of a group of complex diseases of complex etiologies. Some factors are recognized, including genetic factors and lifestyle choices, like smoking, diet and exercise; certain types of infections and the exposition to some chemical substances and radiation ^14^. In Medellin, the majority of cancer determinants are yet to be identified.

Lung cancer is the primary cause of mortality in men and women in Medellin during the five-year period. The prevalence of smoking in the city reaches 25.5%, inversely proportional to the educational level and the proportion of smokers is greater in people that also have a high consumption of alcohol. In Colombia, Medellin is recognized as the city with greatest level of air pollution, exceeding the norms established by the WHO and posing as a risk to human health. Mortality from lung cancer is 2.4 times greater than in Bogota and 1.7 times greater than the mortality in Colombia by the same cause ^15^ ^,^ ^16^.

Different Mexican studies indicate that 50% of women with breast cancer are younger than 50 years old at the time of diagnosis, in contrast to 22% to the Caucasian population ^17^ ^,^ ^18^. In Medellin, the data shows that during 2010-2014, 42.5% of the cases occurred in women younger than 50. This increased proportion of cases in young women is important because the diagnosis and behavior are generally more aggressive, with a disproportionately greater number of years lost due to cancer ^19^ ^,^ ^20^ This is a result of detection at advanced stages, a greater proportion of triple negative tumors with HER2 over-expression and higher rates of systemic relapse in any clinical stage in comparison with postmenopausal women ^21^ ^-^ ^23^.

Prostate cancer was the first cause of morbidity and the second cause of cancer mortality in Medellin, 84.2% was concentrated in the 60 to 80 age group. Prostate cancer affects older men more frequently, which is an important health concern in developed countries (Table 2S). In these countries 15% of cancers in men are prostate cancer in contrast to 4% in developing countries ^24^ ^,^ ^25^. 

Malignant colorectal tumors in Medellin during 2010-2014 affected more women than men. In both sexes, the cases increased after 55 years of age. Colombia can be classified as a country with a low risk for colorectal cancer, but its incidence has increased, coinciding with profound lifestyle changes. The majority of Colombians live in capital cities, few follow the recommendations of exercising a minimum of 150 minutes a week and the prevalence of overweightness is an increasing trend. This condition is more prevalent in women and in the 50 to 64 age group ^26^ ^,^ ^27^.

Gastric cancer is the principle cause of cancer mortality in Colombia ^5^, the risk is greater for men and the age specific rates increase exponentially at 60 years of age (Table 2S and Figure 3). The epidemiology of disease varies considerably by region and sex, due to the difference in eating habits, age and other risk factors in the population ^28^. In Colombia, gastric cancer has an annual incidence of 16.3/100,000 inhabitants and mortality is calculated at 14.2/100,000 inhabitants ^5^. Five-year survival is less than 15% because patients are diagnosed at advanced stages ^29^ ^-^ ^31^ The cause M:I for gastric cancer was greater than 1, suggesting that incidence rates are underestimated (Table 1S). 

Table 2 shows the comparison of the ASIR for all cancer localizations in Medellin with four other Colombian registries situated in Cali ^32^, Bucaramanga ^33^, Manizales ^34^ and Pasto ^35^. The average annual ASR was 144 per 100,000 men and 145 per 100,000 women during 2010-2014. These findings are comparable to those observed in Pasto (134 men and 146 women per 100,000), albeit lower than those in Cali (205 men and 186 women); Bucaramanga (154 men and 157 women) and Manizales (156 men and 165 women per 100,000). A notable difference from other cancer registries is that Medellin has a high percentage of cases by microscopy, which suggests possibly considering a subregister.

**Table 2:** Age standardized incidence rates (world population) of the principle types of cancer. Data comparison among the Population Registries in Colombia.

Localizations	RPC. Incidence Rates 2008-2012								Medellín (RPC-A) 2010-2014	
	Cali^**(**^ ^**32**^ ^**)**^		Bucaramanga^**(**^ ^**33**^ ^**)**^		Manizales ^**(**^ ^**34**^ ^**)**^		Pasto^**(**^ ^**35**^ ^**)**^		
	Men	Women	Men	Women	Men	Women	Men	Women	Men	Women
Stomach	20.3	10.8	17.3	10.3	20.3	9.7	26.5	11.9	12.3	8.1
Prostate	60.1		41.1		44.1		27.1		38.6	
Colon	10.5	9.7	9.5	9.9	8.4	10.0	4.5	5.4	7.0	6.4
Rectum	5.7	4.4	5.0	3.8	6.3	4.7	3.8	3.6	4.7	3.4
Breast		44.5		41.3		37.2		27.8		36.5
Cervix		15.4		12.9		17.5		18.1		8.5
All	205.0	185.7	153.7	156.5	156.0	164.8	134.1	145.6	144.4	145.6
VM%	86.7	89.8	81.5	88.0	85.8	84.7	83.3	84.4	97.4	95.5
DCO%	1.9	1.4	9.1	6.6	3.8	3.0	5.0	4.5	0.2	0.2
M:I¶¶	55.3	53.3	68.3	60.7	74.6	68.8	63.8	52.6	72.3	62.5

Source: ¶ Cancer Incidence in Five Continents, Volume XI. Cancer Incidence in Five Continents, Volume X.

The comparison of the ASIR of PDCC-cancers in Medellin, data provided by the RPC-A, with the ASIR reported by the RPC-Cali, RPC-Bucaramanga, RPC-Manizales and RPC-Pasto shows that the ASIR for breast and prostate cancer were similar to those reported by RPC-Manizales and lower than other Colombian cities. For stomach cancer the ASR in women showed little variation from 8.1 in Medellin and 11.9 in Pasto. In men there was greater contrast, 12.3 in Medellin to 26.5 in Pasto. In Medellin the incidence of colon cancer is lower than those reported in other registries with the exception of Pasto that reported the lowest ASIR in both sexes for this malignant tumor. It is noteworthy that the ASIR for cervical cancer in Medellin is lower than those reported by other registries (Table 2).

In Medellin the incidence rate of cervical cancer is below the national average and the mortality rates are very close to the goals proposed in the PDCC for 2021: Reduce the mortality rate of cervical cancer to 5.5 per 100,000 women. The work of the Sectional Secretary of Health and Social Protection of Antioquia is noteworthy, continuing the cervical cancer detection and control Program that the National Cancerology Institute implemented in Colombia during the 90s and later continued under the auspices of the National Institute of Health in the quality control Program in cervicouterine cytology. The peak of incidence of precursor lesions to cervical cancer in women below 35 is proof that the screening activities are effective in detecting in women in stages previous to invasive cancer (Fig. 2) ^2^ ^,^ ^36^ ^,^ ^37^

Antioquia relies on a consolidated network of cytology laboratories, led by the Public Health Laboratory of the State of Antioquia and by means of the quality control Program, the awareness of undergoing conventional cytology is improving, detecting cervical cancer in pre-invasive stages, which is 100% curable. According to the National Survey of Demographics and Health (or ENDS for its acronym in Spanish) 2015 ^38^ the practice of cytology in Antioquia was 96.2% and the percentage of women who have not undergone a cytology was 3.4%. This number confirms the wide coverage of screening tests in the Antioquian population. 

### Limitations

The low proportion of registered cases based on non-microscopic methods (2.5%) suggests that some cancer patients in the area were not captured and the data is incomplete. The cause M:I is greater than 1 in stomach cancers and in patients older than 70 with prostate and breast cancer. 

### Strengths

The data was obtained based on the population in the nucleus of the municipality of Medellin. The technical and financial guidance was provided by the Sectional Secretary of Health and Social Protection of Antioquia. The project was led by a medical doctor, a pathology specialist, an official of more than 30 years in this institution. 

## Supplementary tables

**Table 1S:** Municipality of Medellin, Colombia. Incidence data of malignant tumors prioritized in the Decade Cancer Control Plan in Colombia during 2010-2014.

Localization	n	Age Desc	0-4	5-9	10-14	15-19	20-24	25-29	30-34	35-39	40-44	45-49	50-54	55-59	60-64	65-69	70-74	75-79	80 +	Crude	ASR
Breast (C50)	3,286	14	0.0	0.0	0.0	0.0	1.2	5.8	16.2	27.7	56.9	91.5	85.5	112.9	123.3	147.5	160.9	141.3	166.6	51.9	36.5
Cervix (C53)	708	1	0.0	0.0	0.0	0.2	1.4	9.0	12.9	20.0	18.0	17.2	17.4	21.2	16.6	20.7	15.0	9.2	19.1	11.2	8.5
Prostate (C61)	2,571	15	0.0	0.0	0.0	0.0	0.0	0.0	0.2	0.9	3.9	12.2	28.6	89.3	187.4	293.1	401.9	411.7	407.7	45.7	38.6
Colorrectal (C18-C20)	1,640	5	0.0	0.0	0.1	0.5	0.6	2.7	2.8	3.8	7.2	10.9	14.7	23.7	37.5	53.9	62.3	87.5	128.8	13.7	10.0
Men	747	3	0.0	0.0	0.0	0.6	0.6	3.1	3.5	2.0	5.2	11.6	14.3	24.1	40.7	66.1	72.4	99.0	149.7	13.3	11.0
Women	893	2	0.0	0.0	0.3	0.4	0.6	2.2	2.2	5.4	8.9	10.3	15.0	23.4	35.1	44.9	55.2	80.2	117.0	14.1	9.4
Colon (C18)	1,080	3	0.0	0.0	0.1	0.3	0.5	2.1	1.6	3.4	4.9	7.1	9.8	14.0	25.4	34.5	40.0	59.4	84.8	9.0	6.6
Men	474	3	0.0	0.0	0.0	0.4	0.6	2.5	1.7	1.7	3.6	7.7	10.4	13.3	27.0	39.5	47.4	60.4	90.6	8.5	7.0
Women	606	0	0.0	0.0	0.3	0.2	0.4	1.8	1.5	4.9	5.9	6.6	9.3	14.6	24.1	30.8	34.8	58.8	81.5	9.6	6.4
Rectum (C19-C20)	560	2	0.0	0.0	0.0	0.2	0.1	0.5	1.2	0.4	2.4	3.8	4.9	9.7	12.2	19.4	22.3	28.1	44.0	4.7	3.4
Men	273	0	0.0	0.0	0.0	0.2	0.0	0.6	1.7	0.3	1.6	3.9	4.0	10.8	13.7	26.5	25.0	38.6	59.1	4.8	4.0
Women	287	2	0.0	0.0	0.0	0.2	0.2	0.4	0.7	0.5	3.0	3.7	5.7	8.8	11.0	14.1	20.4	21.4	35.5	4.6	3.0
Stomach (C16)	1,209	12	0.0	0.0	0.0	0.0	0.4	1.6	3.6	3.8	4.6	7.7	10.4	17.4	25.2	35.8	60.9	66.0	88.0	10.2	10.1
Men	695	6	0.0	0.0	0.0	0.0	0.2	1.9	3.5	4.0	3.6	10.4	14.8	27.2	32.2	47.2	90.5	103.8	123.3	12.4	12.3
Women	514	6	0.0	0.0	0.0	0.0	0.6	1.4	3.7	3.6	5.4	5.4	6.9	9.8	19.7	27.3	40.2	42.0	68.1	8.2	8.1
All localizations	22,379	111	12.0	11.4	11.1	19.1	25.7	48.2	77.2	97.2	125.6	178.8	221.5	345.8	483.8	661.9	874.5	943.1	11,75.0	187.1	143.4
Men	9,602	43	13.9	11.7	11.1	18.0	22.7	33.7	48.6	52.6	71.4	116.8	160.7	309.5	524.3	793.8	1,125.0	1,295.0	15,74.0	171.3	144.4
Women	12,777	68	10.1	11.0	11.0	20.2	28.7	62.1	102.5	134.8	170.8	230.0	270.0	374.5	452.2	563.5	699.9	720.2	950.1	202.9	145.6
Non-invasive cervical neoplasias	2,254	44	0.0	0.0	0.0	4.4	35.3	85.6	99.7	71.1	61.7	37.7	26.2	22.7	25.0	20.3	18.6	6.9	5.0	35.6	30.6
Infantile Leukemia	77	0	3.7	3.2	3.2															0.6	1.1
Men	38	0	3.7	3.4	2.7															0.7	1.0
Women	39	0	3.6	3.0	3.8															0.6	1.1

**Source:** Population-based Cancer Registry of Antioquia.

**Table 2S:** Municipality of Medellin, Colombia. Mortality information of malignant tumors prioritized in the Decade Cancer Control Plan in Colombia during 2010-2014.

Localization	n	0-4	5-9	10-14	15-19	20-24	25-29	30-34	35-39	40-44	45-49	50-54	55-59	60-64	65-69	70-74	75-79	80 +	Crude	ASR
Breast (C50)	1,075	0.0	0.0	0.0	0.2	0.0	1.2	2.6	7.5	11.3	19.1	24.5	27.1	41.6	51.1	73.2	62.6	129.7	17.0	12.9
Cervix (C53)	391	0.0	0.0	0.0	0.0	0.4	2.6	4.2	5.1	7.4	6.7	9.9	8.5	13.8	15.0	20.4	18.3	31.9	6.2	4.9
Prostate (C61)	798	0.0	0.0	0.0	0.0	0.0	0.0	0.2	0.0	0.5	0.2	1.7	7.1	25.4	46.6	99.2	142.4	489.5	14.2	14.6
Colorrectal (C18-C20)	1,216	0.0	0.0	0.1	0.3	0.4	0.9	1.9	1.8	3.7	6.3	8.3	14.0	21.7	37.6	62.3	63.6	142.4	10.2	7.2
Men	575	0.0	0.0	0.2	0.4	0.2	1.5	2.5	1.2	2.9	6.8	9.4	13.6	19.7	53.1	74.2	77.3	173.6	10.2	8.4
Women	641	0.0	0.0	0.0	0.2	0.6	0.4	1.3	2.4	4.3	5.8	7.5	14.4	23.2	26.0	54.0	55.0	124.8	10.1	6.5
Colon (C18)	1015	0.0	0.0	0.1	0.2	0.4	0.7	1.4	1.2	3.1	4.6	6.6	11.6	17.8	31.8	52.4	54.7	123.3	8.5	6.0
Men	476	0.0	0.0	0.2	0.2	0.2	1.0	2.2	0.6	2.6	5.4	7.2	11.4	16.9	44.8	58.6	64.0	148.5	8.5	6.9
Women	539	0.0	0.0	0.0	0.2	0.6	0.4	0.7	1.7	3.5	3.9	6.1	11.7	18.5	22.0	48.0	48.9	109.2	8.5	5.4
Rectum (C19-C20)	201	0.0	0.0	0.0	0.1	0.0	0.2	0.5	0.7	0.6	1.6	1.8	2.5	3.9	5.8	9.9	8.9	19.0	1.7	1.2
Men	99	0.0	0.0	0.0	0.2	0.0	0.4	0.2	0.6	0.3	1.4	2.2	2.2	2.8	8.3	15.5	13.3	25.2	1.8	1.5
Women	102	0.0	0.0	0.0	0.0	0.0	0.0	0.7	0.7	0.9	1.9	1.4	2.7	4.7	4.0	6.0	6.1	15.6	1.6	1.1
Stomach (C16)	1,467	0.0	0.0	0.0	0.0	0.3	1.3	2.9	4.2	4.7	8.2	13.2	16.1	27.5	39.8	71.1	87.9	151.0	12.3	10.3
Men	801	0.0	0.0	0.0	0.0	0.0	1.7	2.2	4.9	4.9	9.8	16.8	22.9	37.5	54.3	99.2	130.4	195.0	14.2	13.6
Women	666	0.0	0.0	0.0	0.0	0.6	1.0	3.5	3.6	4.6	6.9	10.3	10.7	19.7	29.1	51.6	61.1	126.2	10.5	8.0
All localizations	14,922*	5.3	5.3	5.2	10.0	8.1	10.0	20.9	27.3	37.9	65.6	106.3	172.2	282.4	450.0	703.6	897.7	1657.9	124.7	89.7
Men	6,941	4.5	5.5	3.6	12.2	9.0	10.0	17.3	20.5	27.5	58.5	98.3	178.4	318.4	520.7	845.1	1101.0	2129.0	123.3	101.1
Women	7,980	6.2	5.2	6.8	7.8	7.2	10.0	24.1	33.1	46.5	71.4	112.7	167.3	254.4	397.1	605.0	769.1	1391.8	126.0	82.6
Infantile Leukemia	46	1.2	2.3	2.5															0.4	0.6
Men	21	0.5	2.4	2.4															0.4	0.5
Women	25	2.0	2.2	2.5															0.4	0.7

*There is one case of mortality of unknown gender. **Source:** Population-based Cancer Registry of Antioquia.
