# Supplementary material for: Cancer incidence and mortality in Medellin-Colombia, 2010-2014
Source: Colomb Med (Cali). 2018 Mar 30;49(1):81–8. doi: 10.25100/cm.v49i1.3740 (PMC6018825; doi:10.25100/cm.v49i1.3740)
Supplement: Related texts

# Research article: Incidencia y mortalidad por cáncer en Medellín-Colombia, 2010-2014

## Introducción

El cáncer es una de las principales causas de morbilidad y mortalidad en todo el mundo; en 2015 ocasionó 8.8 millones de muertes. Se prevé que el número de nuevos casos aumente en aproximadamente un 70% en los próximos 20 años ^1^^,^^2^. Para el periodo 2007-2011 se estimaron en Colombia 29,734 casos nuevos de cáncer por año en hombres y 33,084 en mujeres. Las tasas de incidencia estandarizadas por edad (TIEE) por cada 100,000 habitantes, para todos los cánceres excepto piel, en hombres fueron de 151.5 y en mujeres, de 145.6 ^3^. 

Para enfrentar este reto, el Ministerio de Salud y la Protección Social y el Instituto Nacional de Cancerología de Colombia (INC) implementaron el *Modelo para el control del cáncer en Colombia* en el 2006. Este modelo define el control del cáncer como un conjunto de actividades que, de forma organizada, se orientan a disminuir la carga de esta enfermedad en Colombia y, para su ejecución, es fundamental describir la situación epidemiológica del cáncer en la población y evaluar los factores determinantes con el propósito de que el sistema de salud oriente los servicios oncológicos y presentar una respuesta social adecuada ^4^. El Plan Decenal para el Control de Cáncer en Colombia (PDCC) ^5^ centra sus acciones en el control del cáncer de mama, cuello uterino, próstata, colorrectal y leucemia aguda pediátrica. Estos tumores, junto con el cáncer de estómago, corresponden al 31.7 % de la mortalidad por cáncer en Colombia para el periodo 2007-2011^3^.

El Registro Poblacional de Cáncer de Antioquia (RPC-A) se encuentra en actividad desde el año 2000 y tiene como misión ofrecer información de calidad y confiable sobre el cáncer en el departamento de Antioquia y facilitar la implementación de los programas de prevención, diagnóstico y manejo integral del cáncer para disminuir la carga de esta enfermedad en la región. En la actualidad, el RPC-A forma parte de la Asociación Internacional de Registros de Cáncer (IACR, por sus siglas en inglés) y de la Red de Registros poblacionales de cáncer de Colombia junto con los RPC de Cali, Bucaramanga, Pasto, Manizales y Barranquilla.

El presente estudio describe la incidencia y mortalidad por cáncer en el municipio de Medellín durante el periodo 2010-2014, como el aporte que hace el RPC-A al conocimiento del comportamiento del cáncer en el departamento y como contribución al logro de los objetivos del PDCC.

## Materiales y Métodos

Medellín es lasegunda ciudad más poblada de Colombia, capital del departamento de Antioquia. Está situada en medio de la Cordillera Central de los Andes a una altitud de 1,538 msnm. El clima es cálido, con poca variación en las temperaturas a lo largo del año. Es la ciudad núcleo del área metropolitana del Valle de Aburrá. Los otros miembros de norte a sur son los municipios de Barbosa, Girardota, Copacabana, Bello, Envigado, Itagüí, Sabaneta, La Estrella y Caldas. La industria representa el 43.6% del producto interno. Los sectores industriales con mayor participación en el valor agregado generado son las textileras, con 20%; sustancias y productos químicos, con el 14.5%; alimentos, con el 10% y bebidas con el 11%. El 10% restante comprende sectores como el metalmecánico, eléctrico y electrónico, entre otros ^6^. 

### Población

Los habitantes descienden de la mezcla de elementos europeos, indígenas y africanos, con un claro predominio de los primeros. Según estimaciones del Departamento Administrativo Nacional de Estadísticas de Colombia (DANE), en el año 2013 el municipio de Medellín contaba con 2,386,233 habitantes en su área urbana y 31,092 en la rural, de los cuales el 52.9% estaba conformado por mujeres (Fig. 1). El Índice de envejecimiento pasó de 8 adultos mayores por cada 100 menores de 15 años en 1964 a 51 adultos mayores en el 2013; la esperanza de vida pasó de 60 años a mediados del siglo XX a 77.5 años en 2013, en hombres 75.5 y mujeres 78.7, respectivamente ^7^.

**Figura 1:** Medellín, Colombia. Pirámide de población, 2013. Fuente: Elaboración basada en las proyecciones de población del Departamento Administrativo Nacional de Estadística. DANE, Colombia, 2013.

### Área de registro

Cabecera del municipio de Medellín con una extensión de 105 km^2^ de suelo urbano y 270 km^2^ de suelo rural.

### Definición de caso

Para el presente estudio, el RPC-A, se comporta como un registro poblacional selectivo. Se incluyeron todos los tumores malignos invasivos diagnosticados por primera vez durante el quinquenio 2010-2014 (incidente) con las localizaciones que fueron priorizados en las líneas estratégicas del PDCC 2012-2020: mama (en mujeres), cuello uterino, próstata, colorrectal; y leucemia aguda pediátrica), además estómago. La base para el diagnóstico fue histológica, citológica de médula ósea, clínica o por certificado de defunción. Para los cálculos estadísticos, se incluyeron tumores malignos primarios únicos o múltiples invasores. En el análisis se tuvieron en cuenta los casos de neoplasias preinvasivas de cuello uterino. Se excluyeron tumores benignos y de comportamiento incierto. Los casos que han llegado a la ciudad para fines de tratamiento o diagnóstico no son considerados residentes de Medellín. La fecha de incidencia correspondió al primer evento cronológico de confirmación diagnóstica de la enfermedad.

La información se recolectó de forma activa y pasiva, continua y sistemática en las instituciones de salud que producen información sobre cáncer: hospitales, clínicas, unidades de oncología, laboratorios de patología y hematología, centros médicos, consultorios especializados, el DANE y la Secretaría de Salud Departamental, organismo encargado de procesar los certificados de defunción, que provee la base de mortalidad oficial. Los datos recolectados se relacionaron con variables sociodemográficas del paciente, clínica del tumor y el seguimiento. La información recolectada se trabajó de acuerdo con los criterios de confidencialidad que impone la IACR para los registros poblacionales de cáncer ^8^^,^^9^.

Los casos se ingresaron al sistema diseñado por el RPC-A para la eliminación de duplicados, procesamiento y complementación de datos. La identificación de tumores múltiples primarios siguió las normas de la IARC ^10^. Se codificó la topografía (localización) y morfología (histología) de los tumores con la Clasificación Internacional de Enfermedades para Oncología, tercera edición (CIE-O-3) ^11^. Para fines comparativos, se agruparon algunas localizaciones. 

Para el control de calidad del RPC-A se emplea un grupo de indicadores, los de exhaustividad (porcentaje de casos incidentes identificados por el certificado de defunción y la razón mortalidad/incidencia), además de indicadores de validez (distribución de casos según la base más válida para el diagnóstico de cáncer y porcentaje de casos de verificación microscópica).

Las tasas de incidencia y mortalidad se calcularon de forma convencional y como denominador las estimaciones y proyecciones poblacionales a mitad de año calculadas a partir del Censo oficial de 2005 ^12^. Se excluyeron los casos sin edad (0.5% 111/22,379) y los tumores *in situ*. Para el caso de cuello uterino, las tasas de incidencia para las neoplasias invasivas y preinvasivas se calcularon por separado. Las tasas estandarizadas por edades se estimaron por el método directo con la población estándar mundial (SEGI), las tasas específicas se calcularon por las variables: género, localización y edad según rangos quinquenales (18 categorías). Los resultados de incidencia y mortalidad se presentan para el periodo 2010-2014 en tablas o gráficos específicos. 

## Resultados

Para el periodo 2010 al 2014 se registraron 56,650 casos nuevos de cáncer en el RPC-A, (60.1 % en mujeres); y 30,465 defunciones por cáncer en el departamento de Antioquia (48.4 % en hombres). El 45.0% de los casos nuevos y 48.9% de las defunciones por cáncer ocurrieron en residentes permanentes del municipio de Medellín. 

De los 22,379 casos nuevos de cáncer diagnosticados en Medellín, 61.6% se presentó en mujeres. La edad promedio al diagnóstico de cáncer en los hombres fue 63 años y en las mujeres 55 años. Las tasas específicas, crudas (TIC) y estandarizadas por edad (TIEE) con la población mundial de referencia se expresan por 100,000 personas-año (pa). En los hombres, las tasas de incidencia de cáncer por 100,000 pa para todas las localizaciones fueron 171.3 (TIC) y 144.4 (TIEE). En las mujeres, la TIC fue 202.9 y la TIEE 145.6. 

En los hombres, las cinco localizaciones principales de cáncer fueron próstata (26.9 %), colorrectal (7.8%), estómago (7.3%), pulmón (6.1%) y vejiga (6.1%). En las mujeres, las localizaciones más frecuentes para cáncer fueron: mama (25.8%), seguido por tiroides (13.6%), colorrectal (7.0%), cuello uterino (5.5%), y pulmón (4.4%). Estas localizaciones representaron en conjunto el 54.7% de todos los casos nuevos de cáncer diagnosticados durante el quinquenio. 

De las 14,922 muertes certificadas por cáncer en Medellín, el 53.4 % ocurrió en mujeres. La edad promedio en el momento de la muerte fue 68 en los hombres y 67.2 en las mujeres. En los hombres, las tasas de mortalidad de cáncer por 100,000 pa para todas las localizaciones fueron 123.3 (TC) y 101.1 (TIEE). En las mujeres, la TIC fue 126.0 y la TIEE 82.6. Las tasas de incidencia y mortalidad por cáncer específicas por edad fueron más altas en las mujeres menores de 55 años, y luego de esta edad, en los hombres. (Tablas suplementarias 1S y 2S).

El cáncer de pulmón (19.0%) fue la primera causa de mortalidad en los hombres, seguidos del cáncer de estómago (11.5%), próstata (11.5%), colorrectal (8.3%) e hígado (6.2%). El cáncer de pulmón fue la principal causa de muerte en mujeres (15.1%), seguido del cáncer de mama (13.5%), estómago (8.3%), colorrectal (8.0%) y leucemias/linfomas (7.6%). En conjunto estas primeras cinco causas, constituyeron el 52.5% de todas las muertes por cáncer ocurridas en Medellín durante el quinquenio 2010 y 2014.

### Cánceres priorizados por el Plan Decenal para el Control del Cáncer en Colombia

Del total de casos de cáncer nuevos diagnosticados en los residentes de Medellín, 9,538 (42.6%) correspondieron al cáncer de estómago, próstata, mama, cuello uterino, colorrectal y leucemia aguda pediátrica que, en conjunto, llamáremos para el análisis cánceres-PDCC; 4,075 (42.7%) casos se diagnosticaron en los hombres y 5,463 (57.3 %) en las mujeres. 

Para el grupo de cánceres-PDCC, la TIC y TIEE por 100,000 pa fue 72.3 y 60.9 en los hombres; y 86.3 y 61.3 en las mujeres. 

Durante el periodo 2010-2014 se diagnosticaron 2,254 mujeres con lesiones preinvasivas de cuello uterino, en 44.0 no se conoció la edad, la TIC fue 35.6 y la TIEE 30.5.

### Criterios de calidad de la información de incidencia

La Tabla 1 muestra los indicadores de calidad de la información de incidencia para el periodo 2010-2014, para los tumores malignos priorizados en el PDCC. El porcentaje de casos diagnosticados con verificación microscópica (histología de tumor primario, citología y aspirado de médula ósea) para hombres y mujeres fue del 99.8%; en este grupo de tumores el porcentaje de casos registrados teniendo como única evidencia el certificado de defunción fue del 0.2%. La razón global de mortalidad: incidencia fue 0,67 para el total de la población, 0.72 en hombres y 0.62 para mujeres.

**Tabla 1:** Municipio de Medellín, Colombia. Datos de incidencia y mortalidad de los tumores malignos priorizados en el Plan Decenal para el control del cáncer en Colombia, Indicadores de calidad (exhaustividad y validez) distribuidos por sexo y localización durante el periodo 2010-2014. Indicadores de calidad de la incidencia.

Localización	Incidencia					M:I	VM%	DCO%	Mortalidad			
	n	%	Edad desc	Tasas					n	%	Tasas	
				Desc	TIC						TIEE	TIC	TIEE
Mama (C50)	3,286	25.7	14	51.9	36.5	0.33	97.4	0.1	1,075	13.5	17.0	12.9
Cuello uterino (C53)	708	5.5	1	11.2	8.5	0.55	98.0	0.1	391	4.9	6.2	4.9
Próstata (C61)	2,571	26.8	15	45.7	38.6	0.31	99.3	0.2	798	11.5	14.2	14.6
Colorrectal (C18-C20)	1,640	7.3	5	13.7	10.0	0.74	98.5	0.2	1,216	8.1	10.2	7.2
Hombre	747	7.8	3	13.3	11.0	0.77	98.4	0.0	575	8.3	10.2	8.4
Mujer	893	7.3	2	14.1	9.4	0.72	98.6	0.3	641	8.0	10.1	6.5
Colon (C18)	1,080	4.8	3	9.0	6.6	0.94	98.6	0.2	1,015	6.8	8.5	6.0
Hombre	474	4.9	3	8.5	7.0	1.00	98.5	0.0	476	6.9	8.5	6.9
Mujer	606	4.7	0	9.6	6.4	0.89	98.7	0.3	539	6.8	8.5	5.4
Recto (C19-C20)	560	2.5	2	4.7	3.4	0.36	98.8	0.2	201	1.3	1.7	1.2
Hombre	273	2.8	0	4.8	4.0	0.36	98.8	0.0	99	1.4	1.8	1.5
Mujer	287	2.2	2	4.6	3.0	0.36	99.3	0.3	102	1.3	1.6	1.1
Estómago (C16)	1,209	5.4	12	10.2	10.1	1.21	98.3	0.4	1,467	9.8	12.3	10.3
Hombre	695	7.2	6	12.4	12.3	1.15	98.4	0.3	801	11.5	14.2	13.6
Mujer	514	4.0	6	8.2	8.1	1.30	98.1	0.6	666	8.3	10.5	8.0
Todas las localizaciones‡	22,379		111	187.1	143.4	0.67	96.3	0.2	14,922		124.7	89.7
Hombres	9,602	42.9	43	171.3	144.4	0.72	97.4	0.2	6,941		123.3	101.1
Mujeres	12,777	57.1	68	202.9	145.6	0.62	95.5	0.2	7,980		126.0	82.6
Leucemias Infantiles	77		0	0.6	1.1	0.60	98.7	1.3	46		0.4	0.6
Hombre	38		0	0.7	1.0	0.55	98.8	1.2	21		0.4	0.5
Mujer	39		0	0.6	1.1	0.64	98.6	1.4	25		0.4	0.7

Fuente: Registro Poblacional de Cáncer de Antioquia. Estadísticas Vitales SSSA- DANE

n: número de casos;

M:I : Razón Mortalidad-Incidencia;

VM: Verificación microscópica (histología/hematología/citología);

DCO: Solo certificado de defunción. Las tasas específicas, crudas (TIC) y estandarizadas por edad (TIEE) con la población mundial se expresan por 100,000 pa.

‡ Todas las localizaciones, excepto C44 (Piel no melanoma)

#### Cáncer de mama

La edad promedio del diagnóstico fue de 58 años, el 42.5% ocurrió en menores de 50 años y solo el 12.5% en mayores de 80 años (Fig 2).

**Figura 2:** Medellín, Colombia, 2010-2014. Tasas de incidencia específicas por edad y sexo (por 100,000 pa) para los 9,538 cánceres priorizados en el Plan Decenal para el Control del Cáncer en Colombia. **A.** Las tasas de incidencia específicas por edad fueron más altas en cáncer de próstata y más bajas en cáncer de cuello uterino. El pico de lesiones precursoras de cáncer de cuello uterino se presentó alrededor de los 40 años. **B.** La morbilidad por cáncer gástrico fue más alta en los hombres mayores de 45 años, en cáncer colorrectal la diferencia fue menos aparente con ligero predominio en mujeres menores de 50 años. **Fuente:** Registro Poblacional de Cáncer de Antioquia.

#### Cáncer de cérvix

Las tasas de incidencia específicas por edad de cáncer invasor de cuello uterino alcanzaron el valor máximo de 20 por 100,000 pa alrededor de los 30 años y luego hacen una meseta y permanecen estables en todos los grupos de edad, la mitad de los casos se diagnosticó antes de los 50 años y únicamente el 4.3% en mayores de 80 años. 

#### Neoplasias preinvasivas de cuello uterino

El pico máximo de incidencia de las lesiones preinvasivas de cérvix fue entre los 35 y 39 años, el 60.8 % se diagnosticó antes de los 40 años. 

#### Cáncer de próstata

la edad promedio al diagnóstico fue de 68 años, de 65 años para cáncer colorrectal y cáncer de estómago y 7 años para leucemia aguda pediátrica (Fig. 2).

### Mortalidad por tumores malignos priorizados en el PDCC en Medellín

Durante el periodo 2010-2014, los cánceres-PDCC representaron el 33.5 % (4,993) del total de las muertes por cáncer en Medellín (14.922); 2.798 (56.0 %) de las defunciones ocurrieron en mujeres y 2,195 (44.0 %) en hombres.

La tasa de mortalidad cruda (TMC) y estandarizada (TMEE) por edad para el grupo de cánceres-PDCC; fue 38.9 y 31.6 en los hombres; y 44.2 y 29.7 en las mujeres.

La edad promedio en el momento de la muerte fue de 64 años para cáncer de mama, 58 años para cáncer de cuello uterino, 78 años para cáncer de próstata, 68 años para cáncer colorrectal y cáncer de estómago. Por su parte, en la leucemia aguda pediátrica, la edad promedio de muertes fue de nueve años. 

La Figura 3 describe las curvas de las tasas de mortalidad específicas por edad. La mortalidad por cáncer de mama es más alta en todas *las personas menores de 65 años. En adultos mayores de 65 años las muertes son ocasionadas por el cáncer de próstata y estómago. La* razón (M:I) es mayor a uno, en las mujeres con cáncer de cuello uterino y en los hombres con cáncer de próstata mayores a 70 años.

**Figura 3:** Medellín, Colombia, 2010-2014. Tasas de mortalidad específicas por edad y sexo (por 100,000 pa). Fuente: Registro Poblacional de Cáncer de Antioquia y Estadísticas vitales SSSA- DANE.

Las tasas específicas de mortalidad por debajo de los 70 años son más altas en las mujeres con cáncer de mama. Después de los 70 años la razón M:I es mayor a uno en los hombres con cáncer de próstata y en las mujeres con cáncer de cuello uterino.

## Discusión

El Registro Poblacional de Cáncer de Antioquia (RPC-A) recopiló y clasificó todos los casos nuevos de cáncer y las defunciones por cáncer ocurridas en Medellín, la segunda ciudad más poblada de Colombia. Esta comunicación presenta las tasas de incidencia y mortalidad para los cánceres priorizados por el PDCC durante el periodo 2010-2014. Los cánceres-PDCC fueron responsables del 42.4 % de la morbilidad y el 33.4% de la mortalidad por cáncer en Medellín. Este informe es producto del trabajo colaborativo entre la Secretaria de Salud de Antioquia y la red de prestadores de servicios oncológicos de la ciudad. 

La información suministrada por el RPC-A nos indica que el cáncer es un problema de salud pública en Medellín, fue la segunda causa de muerte después de las enfermedades del sistema circulatorio y responsable del 25% de las muertes ocurridas en la ciudad ^13^. Durante el quinquenio (2010-2014) ocasionó en promedio 5,225 casos nuevos y 2,963 defunciones al año en residentes de la ciudad de Medellín, según información suministrada por la Secretaría Seccional de Salud y Protección Social de Antioquia.

El riesgo de morbimortalidad por cáncer en una región se encuentra determinado por múltiples factores. El cáncer comprende un grupo complejo de enfermedades de etiología compleja, algunos de los factores son reconocidos e incluyen factores genéticos y del estilo de vida tal como el tabaquismo, la alimentación y el ejercicio; ciertos tipos de infecciones y la exposición a algunas sustancias químicas y radiaciones ^14^. En Medellín, la mayoría de los determinantes del cáncer se encuentran pendientes de identificar.

El cáncer de pulmón fue la primera causa de mortalidad por cáncer en hombres y mujeres de Medellín durante el quinquenio estudiado. La prevalencia de tabaquismo en la ciudad alcanza el 25.5%, se asocia de manera inversa con el nivel de estudios y la proporción de fumadores es mayor en quienes presentan alto consumo de alcohol. En Colombia, Medellín se reconoce como una de las ciudades con mayor nivel de descargas de contaminantes a la atmósfera y los niveles de contaminación exceden la norma establecida por la OMS como de riesgo para la salud humana. La mortalidad por cáncer de pulmón en Medellín es 2.4 veces mayor que en Bogotá y 1.7 veces mayor que la mortalidad de Colombia por la misma causa ^15^^,^^16^.

En distintas series mexicanas se informa que el 50% de las mujeres con cáncer de mama tiene una edad menor de 50 años al diagnóstico, en contraste con el 22% en la población caucásica ^17^^,^^18^. Para Medellín encontramos que para el periodo 2010-2014, el 42.5 % de los casos ocurrieron en mujeres menores de 50 años. La importancia de esta proporción de casos en mujeres jóvenes radica en que su diagnóstico y comportamiento son generalmente más agresivos, con un número desproporcionado de años de vida perdidos por causa del cáncer ^19^^,^^20^. Esto se debe a la frecuente presentación al diagnóstico en etapas avanzadas, a una mayor proporción de tumores triples negativos y con sobreexpresión de HER2, y a mayores tasas de recaídas sistémicas en cualquier estadio clínico en comparación con las mujeres postmenopáusicas ^21^^-^^23^.

El cáncer de próstata fue la primera causa de morbilidad y la segunda causa de mortalidad por cáncer en Medellín. El 84.2 % se concentró en el grupo de 60 a 80 años. El cáncer de próstata afecta más frecuentemente a los varones de mayor edad, lo que constituye una importante preocupación de salud en los países desarrollados (Tabla 1S). En estos países 15 % de los cánceres en varones es por cáncer de próstata, en comparación con 4% de los casos de cáncer en varones en países en vías de desarrollo ^24^^,^^25^.

Los tumores malignos colorrectales, en Medellín, para el periodo 2010-2014, afectaron más a mujeres que a hombres. En ambos sexos, los casos aumentaron a partir de los 55 años. Colombia puede catalogarse como una población de bajo riesgo para cáncer colorrectal, pero su incidencia ha aumentado y coincide con profundos cambios en el estilo de vida. La mayoría de los colombianos vive en las cabeceras municipales, pero pocos cumplen con las recomendaciones de realizar mínimo 150 minutos de actividad física durante la semana y la prevalencia de exceso de peso presenta una tendencia ascendente. Esta condición es más prevalente en las mujeres y en el grupo de edad de 50 a 64 ^26^^,^^27^.

El cáncer gástrico es la principal causa de mortalidad por cáncer en Colombia ^5^, el riesgo es mayor en los hombres y las tasas específicas por edad aumentan de manera exponencial a partir de los 60 años (Tabla 2S y Fig. 3). La epidemiología de la enfermedad varía considerablemente por región y por sexo, debido a la diferencia en hábitos alimenticios, edad y otros factores de riesgo de la población ^28^. En Colombia, el cáncer gástrico tiene una incidencia anual de 16.3/100,000 habitantes y una mortalidad calculada de 14.2/100,000 habitantes ^5^. La supervivencia a cinco años es inferior al 15 % porque los pacientes llegan en estadios avanzados en el momento del diagnóstico ^29^^-^^31^. La razón M:I para cáncer gástrico fue mayor a uno y sugiere que las tasas de incidencia están subestimadas (Tabla 1).

La Tabla 2 muestra la comparación de las TIEE para todas las localizaciones de cáncer en Medellín, con otros cuatro registros colombianos ubicados en Cali ^32^, Bucaramanga ^33^, Manizales _^34^_ y Pasto ^34^. La TIEE promedio anual fue de 144 por cada 100,000 hombres y de 145 por cada 100,000 mujeres en el periodo 2010-2014. Estos resultados son comparables con los observados en Pasto (134 varones, 146 mujeres por 100,000) aunque más bajos que en Cali (205 varones y 186 mujeres); Bucaramanga (154 varones y 157 mujeres) y Manizales (156 varones y 165 mujeres por 100,000). A diferencia de los otros registros de cáncer, Medellín tiene un alto porcentaje de casos por microscopia lo que sugiere considerar un subregistro (Tabla 2).

**Tabla 2:** Tasa de incidencia estandarizada por edad (población mundial) de los principales tipos de cáncer. Comparación de datos de los Registros poblacionales de Colombia.

Localizaciones	RPC. Tasas de Incidencia 2008-2012¶								Medellín (RPC-A) 2010-2014	
	Cali ^30^		Bucaramanga (31)		Manizales^34^		Pasto^32^		
	Hombres	Mujeres	Hombres	Mujeres	Hombres	Mujeres	Hombres	Mujeres	Hombres	Mujeres
Estómago	20.3	10.8	17.3	10.3	20.3	9.7	26.5	11.9	12.3	8.1
Próstata	60.1		41.1		44.1		27.1		38.6	
Colon	10.5	9.7	9.5	9.9	8.4	10.0	4.5	5.4	7.0	6.4
Recto	5.7	4.4	5.0	3.8	6.3	4.7	3.8	3.6	4.7	3.4
Mama		44.5		41.3		37.2		27.8		36.5
Cuello uterino		15.4		12.9		17.5		18.1		8.5
Todas	205.0	185.7	153.7	156.5	156.0	164.8	134.1	145.6	144.4	145.6
VM %	86.7	89.8	81.5	88.0	85.8	84.7	83.3	84.4	97.4	95.5
DCO %	1.9	1.4	9.1	6.6	3.8	3.0	5.0	4.5	0.2	0.2
M:I¶¶	55.3	53.3	68.3	60.7	74.6	68.8	63.8	52.6	72.3	62.5

Fuente: ¶ Cancer Incidence in Five Continents, Volume XI. Cancer Incidence in Five Continents, Volume X.

Al comparar las TIEE de los cánceres-PDCC en el municipio de Medellín, datos que provienen del RPC-A, con las TIEE reportados por los RPC-Cali, RPC-Bucaramanga, RPC-Manizales y RPC-Pasto, se encontró que, para Medellín, la TIEE para cáncer de mama y próstata fueron similares a las reportadas por el RPC-Manizales y más bajas que las otras ciudades colombianas. Para cáncer de estómago, las TAE en las mujeres mostraron poca variación desde 8.1 en Medellín a 11.9 en Pasto. En los hombres hubo mayor contraste desde 12.3 en Medellín a 26.5 en Pasto. En Medellín la incidencia es más baja que la que reportaron los otros Registros para cáncer colorrectal, excepto al comparar nuestros datos con los de Pasto, que reportó para este tumor maligno una TIEE más baja en ambos sexos. Llama la atención la TIEE para cáncer de cuello uterino, que es más baja en Medellín que la reportada por los otros Registros (Tabla 2).

En Medellín, las tasas de incidencia del cáncer de cuello uterino están por debajo del promedio nacional y las tasas de mortalidad muy cercanas a las metas del PDCC de Colombia para 2021: Reducir la tasa de mortalidad por cáncer de cuello uterino a 5.5 por 100,000 mujeres. Se resalta el esfuerzo de la Secretaría Seccional de Salud y Protección Social de Antioquia, por continuar el Programa de detección y control del cáncer de cuello uterino, que el Instituto Nacional de Cancerología implementó en Colombia desde la década de los 90, y luego se siguió con el Instituto Nacional de Salud con el Programa de control de calidad a la citología cervicouterina. El pico de incidencia de lesiones precursoras de cáncer de cuello uterino en mujeres menores de 35 años de edad es la evidencia de las actividades realizadas para que estas mujeres hayan sido detectadas en estadios previos al cáncer invasivo (Fig. 2) ^4^^,^^35^^,^^36^.

En el departamento de Antioquia se cuenta con una Red consolidada de laboratorios de citología, liderada por el Laboratorio departamental de salud pública de Antioquia y mediante el Programa de control de calidad, se mejora la sensibilidad de la citología convencional y se detecta, mediante esta prueba, el cáncer de cuello uterino en etapas preinvasoras, es decir, el 100 % es curable. La práctica de citologías para el departamento de Antioquia, según la Encuesta Nacional de Demografía y Salud (ENDS) 2015 ^37^ fue del 96.2% y la proporción de mujeres que nunca se ha realizado la citología fue del 3.4 %. Esta cifra confirma la cobertura de la tamización con citologías en la población antioqueña. 

### Limitaciones 

La baja proporción de casos registrados con base en métodos no microscópicos (2.5 %) sugiere que algunos de los pacientes con cáncer en el área no son captados y los datos son incompletos. La razón M: I es mayor a uno en cáncer de estómago y en los pacientes mayores a 70 años con cáncer de próstata y mama. 

### Fortalezas

Los datos fueron obtenidos con base en la población de la cabecera del municipio de Medellín. La dirección técnica y financiera la hace la Secretaría Seccional de Salud y Protección Social de Antioquia. El proyecto lo lidera un médico especialista en Patología, funcionario desde hace 30 años en esta dependencia.

## Tablas suplementarias

**Tabla 1S:** Municipio de Medellín, Colombia. Datos de incidencia de los tumores malignos priorizados en el Plan Decenal para el control del cáncer en Colombia, durante el periodo 2010-2014

Localización	n	Edad desc	0-4	5-9	10-14	15-19	20-24	25-29	30-34	35-39	40-44	45-49	50-54	55-59	60-64	65-69	70-74	75-79	80 +	Cruda	ASR
Mama (C50)	3,286	14	0.0	0.0	0.0	0.0	1.2	5.8	16.2	27.7	56.9	91.5	85.5	112.9	123.3	147.5	160.9	141.3	166.6	51.9	36.5
Cuello del útero (C53)	708	1	0.0	0.0	0.0	0.2	1.4	9.0	12.9	20.0	18.0	17.2	17.4	21.2	16.6	20.7	15.0	9.2	19.1	11.2	8.5
Próstata (C61)	2,571	15	0.0	0.0	0.0	0.0	0.0	0.0	0.2	0.9	3.9	12.2	28.6	89.3	187.4	293.1	401.9	411.7	407.7	45.7	38.6
Colorrectal (C18-C20)	1,640	5	0.0	0.0	0.1	0.5	0.6	2.7	2.8	3.8	7.2	10.9	14.7	23.7	37.5	53.9	62.3	87.5	128.8	13.7	10.0
Hombre	747	3	0.0	0.0	0.0	0.6	0.6	3.1	3.5	2.0	5.2	11.6	14.3	24.1	40.7	66.1	72.4	99.0	149.7	13.3	11.0
Mujer	893	2	0.0	0.0	0.3	0.4	0.6	2.2	2.2	5.4	8.9	10.3	15.0	23.4	35.1	44.9	55.2	80.2	117.0	14.1	9.4
Colon (C18)	1,080	3	0.0	0.0	0.1	0.3	0.5	2.1	1.6	3.4	4.9	7.1	9.8	14.0	25.4	34.5	40.0	59.4	84.8	9.0	6.6
Hombre	474	3	0.0	0.0	0.0	0.4	0.6	2.5	1.7	1.7	3.6	7.7	10.4	13.3	27.0	39.5	47.4	60.4	90.6	8.5	7.0
Mujer	606	0	0.0	0.0	0.3	0.2	0.4	1.8	1.5	4.9	5.9	6.6	9.3	14.6	24.1	30.8	34.8	58.8	81.5	9.6	6.4
Recto (C19-C20)	560	2	0.0	0.0	0.0	0.2	0.1	0.5	1.2	0.4	2.4	3.8	4.9	9.7	12.2	19.4	22.3	28.1	44.0	4.7	3.4
Hombre	273	0	0.0	0.0	0.0	0.2	0.0	0.6	1.7	0.3	1.6	3.9	4.0	10.8	13.7	26.5	25.0	38.6	59.1	4.8	4.0
Mujer	287	2	0.0	0.0	0.0	0.2	0.2	0.4	0.7	0.5	3.0	3.7	5.7	8.8	11.0	14.1	20.4	21.4	35.5	4.6	3.0
Estómago (C16)	1,209	12	0.0	0.0	0.0	0.0	0.4	1.6	3.6	3.8	4.6	7.7	10.4	17.4	25.2	35.8	60.9	66.0	88.0	10.2	10.1
Hombre	695	6	0.0	0.0	0.0	0.0	0.2	1.9	3.5	4.0	3.6	10.4	14.8	27.2	32.2	47.2	90.5	103.8	123.3	12.4	12.3
Mujer	514	6	0.0	0.0	0.0	0.0	0.6	1.4	3.7	3.6	5.4	5.4	6.9	9.8	19.7	27.3	40.2	42.0	68.1	8.2	8.1
Todas las localizaciones	22,379	111	12.0	11.4	11.1	19.1	25.7	48.2	77.2	97.2	125.6	178.8	221.5	345.8	483.8	661.9	874.5	943.1	11,75.0	187.1	143.4
Hombres	9,602	43	13.9	11.7	11.1	18.0	22.7	33.7	48.6	52.6	71.4	116.8	160.7	309.5	524.3	793.8	1,125.0	1,295.0	15,74.0	171.3	144.4
Mujeres	12,777	68	10.1	11.0	11.0	20.2	28.7	62.1	102.5	134.8	170.8	230.0	270.0	374.5	452.2	563.5	699.9	720.2	950.1	202.9	145.6
Neoplasias no invasivas del cuello del útero	2,254	44	0.0	0.0	0.0	4.4	35.3	85.6	99.7	71.1	61.7	37.7	26.2	22.7	25.0	20.3	18.6	6.9	5.0	35.6	30.6
Leucemias infantiles	77	0	3.7	3.2	3.2															0.6	1.1
Hombre	38	0	3.7	3.4	2.7															0.7	1.0
Mujer	39	0	3.6	3.0	3.8															0.6	1.1

**Fuente:** Registro Poblacional de Cáncer de Antioquia.

**Tabla 2S:** Municipio de Medellín, Colombia. Datos de mortalidad de los tumores malignos priorizados en el Plan Decenal para el control del cáncer en Colombia, durante el periodo 2010-2014

Localización	n	0-4	5-9	10-14	15-19	20-24	25-29	30-34	35-39	40-44	45-49	50-54	55-59	60-64	65-69	70-74	75-79	80 +	Cruda	ASR
Mama (C50)	1,075	0.0	0.0	0.0	0.2	0.0	1.2	2.6	7.5	11.3	19.1	24.5	27.1	41.6	51.1	73.2	62.6	129.7	17.0	12.9
Cuello del útero (C53)	391	0.0	0.0	0.0	0.0	0.4	2.6	4.2	5.1	7.4	6.7	9.9	8.5	13.8	15.0	20.4	18.3	31.9	6.2	4.9
Próstata (C61)	798	0.0	0.0	0.0	0.0	0.0	0.0	0.2	0.0	0.5	0.2	1.7	7.1	25.4	46.6	99.2	142.4	489.5	14.2	14.6
Colorrectal (C18-C20)	1,216	0.0	0.0	0.1	0.3	0.4	0.9	1.9	1.8	3.7	6.3	8.3	14.0	21.7	37.6	62.3	63.6	142.4	10.2	7.2
Hombre	575	0.0	0.0	0.2	0.4	0.2	1.5	2.5	1.2	2.9	6.8	9.4	13.6	19.7	53.1	74.2	77.3	173.6	10.2	8.4
Mujer	641	0.0	0.0	0.0	0.2	0.6	0.4	1.3	2.4	4.3	5.8	7.5	14.4	23.2	26.0	54.0	55.0	124.8	10.1	6.5
Colon (C18)	1015	0.0	0.0	0.1	0.2	0.4	0.7	1.4	1.2	3.1	4.6	6.6	11.6	17.8	31.8	52.4	54.7	123.3	8.5	6.0
Hombre	476	0.0	0.0	0.2	0.2	0.2	1.0	2.2	0.6	2.6	5.4	7.2	11.4	16.9	44.8	58.6	64.0	148.5	8.5	6.9
Mujer	539	0.0	0.0	0.0	0.2	0.6	0.4	0.7	1.7	3.5	3.9	6.1	11.7	18.5	22.0	48.0	48.9	109.2	8.5	5.4
Recto (C19-C20)	201	0.0	0.0	0.0	0.1	0.0	0.2	0.5	0.7	0.6	1.6	1.8	2.5	3.9	5.8	9.9	8.9	19.0	1.7	1.2
Hombre	99	0.0	0.0	0.0	0.2	0.0	0.4	0.2	0.6	0.3	1.4	2.2	2.2	2.8	8.3	15.5	13.3	25.2	1.8	1.5
Mujer	102	0.0	0.0	0.0	0.0	0.0	0.0	0.7	0.7	0.9	1.9	1.4	2.7	4.7	4.0	6.0	6.1	15.6	1.6	1.1
Estómago (C16)	1,467	0.0	0.0	0.0	0.0	0.3	1.3	2.9	4.2	4.7	8.2	13.2	16.1	27.5	39.8	71.1	87.9	151.0	12.3	10.3
Hombre	801	0.0	0.0	0.0	0.0	0.0	1.7	2.2	4.9	4.9	9.8	16.8	22.9	37.5	54.3	99.2	130.4	195.0	14.2	13.6
Mujer	666	0.0	0.0	0.0	0.0	0.6	1.0	3.5	3.6	4.6	6.9	10.3	10.7	19.7	29.1	51.6	61.1	126.2	10.5	8.0
Todas las localizaciones	14,922*	5.3	5.3	5.2	10.0	8.1	10.0	20.9	27.3	37.9	65.6	106.3	172.2	282.4	450.0	703.6	897.7	1657.9	124.7	89.7
Hombres	6,941	4.5	5.5	3.6	12.2	9.0	10.0	17.3	20.5	27.5	58.5	98.3	178.4	318.4	520.7	845.1	1101.0	2129.0	123.3	101.1
Mujeres	7,980	6.2	5.2	6.8	7.8	7.2	10.0	24.1	33.1	46.5	71.4	112.7	167.3	254.4	397.1	605.0	769.1	1391.8	126.0	82.6
Leucemias infantiles	46	1.2	2.3	2.5															0.4	0.6
Hombre	21	0.5	2.4	2.4															0.4	0.5
Mujer	25	2.0	2.2	2.5															0.4	0.7

*Existe un caso de mortalidad con género sin identificar.

**Fuente:** Registro Poblacional de Cáncer de Antioquia
